# Quantifying surgical completeness in patients with aspirin exacerbated respiratory disease

**DOI:** 10.1186/s40463-023-00682-1

**Published:** 2023-12-17

**Authors:** Marc Levin, Yvonne Chan, Doron D. Sommer, Andrew Thamboo, John M. Lee

**Affiliations:** 1 Division of Rhinology, Department of Otolaryngology – Head and Neck Surgery, St. Michael’s Hospital, Unity Health Toronto, University of Toronto, 30 Bond Street, 8 Cardinal Carter, Toronto, ON M5B 1WB Canada; 2 Department of Surgery – Division of Otolaryngology Head and Neck Surgery, McMaster University, Hamilton, ON Canada; 3 Department of Surgery – Division of Otolaryngology Head and Neck Surgery, University of British Columbia, Vancouver, BC Canada

**Keywords:** Aspirin exacerbated respiratory disease, Chronic rhinosinusitis, CT scan, endoscopic sinus surgery, Surgical completeness

## Abstract

**Background:**

Aspirin exacerbated respiratory disease (AERD) in patients who have had sinus surgery remains a management challenge. Aspirin desensitization and biologics are additional treatment options. It remains unclear if patients require a more comprehensive surgery prior to implementing such additional therapies. The purpose of this study was to quantify prior surgery completeness in AERD patients at a tertiary rhinology practice.

**Methods:**

Paranasal sinus CT scans were reviewed by four academic rhinologists to assess surgery completeness. Using a published CT grading system, each sinus was graded on the completeness of surgery and middle turbinate reduction. A score out of 14 was calculated for each patient (7 per side).

**Results:**

Sixty-one patients with AERD out of 141 available were included. Mean inter-rater agreement across all sinuses was moderate (k = 0.42). The mean completeness score was 6.7/14. The following procedures were rated as complete (means): uncinectomy (L: 84%, R: 82%, k = 0.44), maxillary (L: 83%, R: 77%, k = 0.32), middle turbinate reduction (L: 45%, R: 46%, k = 0.31), anterior ethmoid (L: 35%, R: 39%, k = 0.51), sphenoid (L: 36%, R: 35%, k = 0.4), posterior ethmoid (L: 30%, R: 30%, k = 0.48), frontal (L: 22%, R: 21%, k = 0.46).

**Conclusion:**

Prior surgery in AERD patients were mostly deemed incomplete. Uncinectomy and maxillary antrostomy are the most common procedures previously performed. It remains toe seen whether this would be considered ‘adequate’ surgery or more ‘complete’ surgery is required to achieve greater disease control.

## Background

Aspirin exacerbated respiratory disease (AERD), classically known as “Samter’s triad”, is characterised by the clinical combination of nasal polyps, bronchial asthma, and aspirin intolerance [1]. The prevalence of AERD is 0.6% to 2.5% in the general population [2–4]. The natural history of AERD is characterised by persistent upper and lower respiratory symptoms despite appropriate therapy including avoidance of ASA or NSAIDs [5].

AERD tends to be more recalcitrant to medical and surgical interventions when compared to other forms of chronic rhinosinusitis (CRS) and thus patients with this diagnosis often require long term medical management and multiple sinus surgeries to control their symptoms [5, 6]. The recalcitrant nature of the disease makes endoscopic sinus surgery (ESS) an important therapy to improve clinical outcomes in addition to the medical therapy. Studies have demonstrated that in the AERD population there is a shorter time to revision surgery and a greater number of revision surgeries needed when compared to patients with other forms of chronic rhinosinusitis (CRS) [7, 8]. For these patients who have refractory disease, new drug therapies in the form of biologics (eg. omalizumab, mepolizumab, and dupilumab) can now be offered for as an alternative/adjunctive treatment. In addition, aspirin desensitization (AD) following sinus surgery has been shown to decrease revision surgery rates and provide more durable long-term symptom outcomes [9]. However, both adjunctive treatments are not without their own risks, costs and require a long-term commitment from patients.

The decision to proceed with additional therapies such as biologics or AD partly relies on the certainty that all other treatment option have been adequately exhausted. To that end, we investigated the completeness of prior sinus surgery in symptomatic AERD patients presenting to a tertiary rhinology clinic. By answering this question, we hope to provide greater clarity in the role of “complete” sinus surgery for this challenging patient population.

## Materials and methods

This study is a retrospective electronic medical chart review of patients with diagnosis of AERD or Samter’s triad at St. Michael’s Hospital (Toronto, Ontario, Canada) between 01 January 2009 and 31 December 2020. Unity Health Toronto Research Ethics approval was granted (REB# 19-104).

Patients included in this study were over 18 years of age and had a diagnosis of AERD based on: (1) Documented nasal polyposis on either endoscopic examination or CT scan, (2) Previous diagnosis and active management of asthma and (3) History of sensitivity reaction to any COX-1 inhibitor (clear history of previous asthma exacerbation or naso-ocular reaction within 3 h of ingestion of COX-1). (4) History of prior sinus surgery.

Patients were excluded if they were: immunocompromised, had cystic fibrosis, allergic fungal sinusitis, negative aspirin challenge, lung disease other than asthma, insufficient information on chart review or did have not a history of sinus surgery. Extended procedures such as endoscopic modified Lothrop procedure and medial maxillectomy were excluded as the purpose was to determine adequacy of prior standard ESS techniques.

Computed tomography (CT) scans that were available and of sufficient quality for each eligible patient were independently reviewed by four academic rhinology surgeons who were blinded to the patient histories. These CT scans were completed after their most recent surgery prior to referral. Each patient’s scan was graded using a previously published CT surgery score grading system [10]. The scans were graded on both the left and right sides in terms of: adequacy of reduction of middle turbinate (1 = yes, 0 = no), completion of uncinectomy (1 = yes, 0 = no) and whether or not there was adequate opening of the frontal (1 = yes, 0 = no), maxillary (1 = yes, 0 = no), anterior ethmoid (1 = yes, 0 = no), posterior ethmoid (1 = yes, 0 = no) and sphenoid sinuses (1 = yes, 0 = no)Each patient received a total bilateral surgical completeness score out of 14. Additionally, patient demographic data was collected including age and gender. Lund-Mackay (LM) and endoscopy scores were also collected [11].

Prior to initiation of data collection, authors collectively agreed upon the definition of adequate opening of each sinus on CT scan. Consensus was achieved that adequate meant that there would be minimal to no additional bony dissection the reviewer would perform during a revision surgery. The presence of polyps/mucosal thickening was not part of the evaluation of adequate surgical opening.

Descriptive statistics were calculated using Microsoft Excel (version 16.59). Fleiss Multi-rater Kappa agreement scores [12] were calculated using SPSS (IBM ®).

## Results

Sixty-one patients (out of 141 available) with AERD were included. We excluded patients who did not meet the inclusion criteria, whose scans were not available or who did not have prior surgery. Twenty-seven of the patients were male and 34 were female. The age range of patients was 24–77 years with a mean of 57 years. The mean LM score for all patients was: 20.1.

**Fig. 1:**
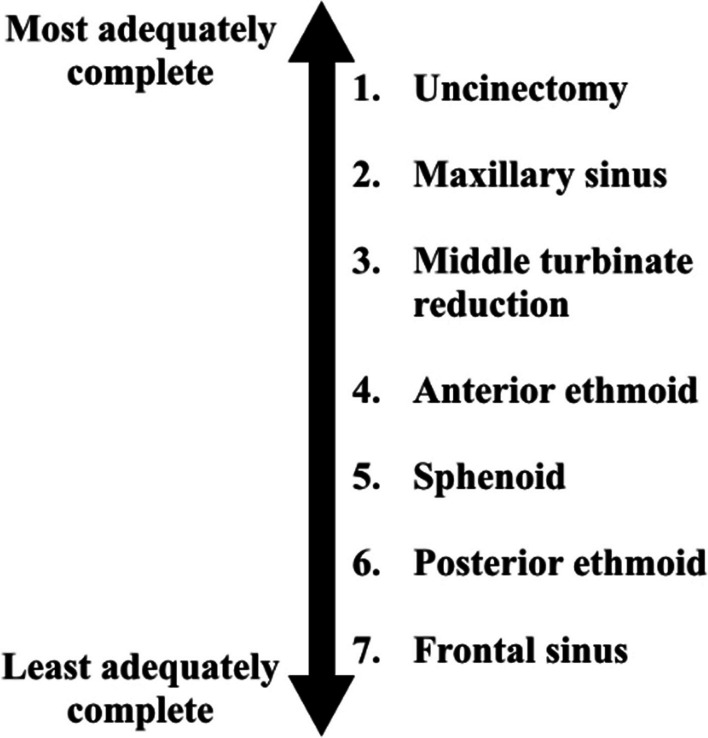
Ranking of adequacy of completeness of surgery by sinus across all reviewers

The mean total completeness score across all patients was 6.7/14 for all reviewers. The highest patient completeness score was 14. The lowest patient completeness score was 1. With regards to uncinectomies, the reviewers rated them as complete in 84% (Left) and 82% (Right) of patients. The reviewers rated the maxillary sinuses as adequately opened in 83% (Left) and 77% (Right) of patients. The middle turbinate reduction was completed in 45% (Left) and 46% (Right) of patients. Adequate opening of the anterior ethmoid was completed in 35% (Left) and 39% (Right) of patients. Adequate opening of the sphenoid was completed in 36% (Left) and 35% (Right) of patients. Adequate opening of the posterior ethmoid was completed in 30% (Left) and 30% (Right) in patients. Finally, frontal opening was completed in 22% (Left) and 21% (Right) of patients (Fig. 1).

Fleiss multi-rater agreement scores were calculated for the mean of all sinuses, k = 0.418. The agreement score for the total bilateral scores was k = 0.121. The detailed agreement scores in each sinus can be found in Table 1.

**Table 1:** Fleiss multi-rater agreement scores

Sinus	Fleiss multi-rater agreement score (k)
Right frontal	0.427
Left frontal	0.493
Right maxillary	0.289
Left maxillary	0.348
Right uncinectomy	0.407
Left uncinectomy	0.481
Right middle turbinate	0.254
Left middle turbinate	0.371
Right anterior ethmoid	0.515
Left anterior ethmoid	0.513
Right posterior ethmoid	0.485
Left posterior ethmoid	0.470
Right sphenoid	0.416
Left sphenoid	0.384
Total scores	0.121
Mean sinus score	0.418

## Discussion

This study was the first to quantify adequacy of prior surgical completeness among symptomatic AERD patients presenting to a tertiary rhinology clinic. Most referrals included patients who had their surgery at non-tertiary healthcare centres prior to referral. This does not necessarily mean however, that their surgeon was not rhinology fellowship trained or experienced in sinus surgery. Not unexpectedly, uncinectomies and maxillary antrostomies were completed in most patients. However, four independent fellowship trained rhinologists who were blinded to the patient histories concluded that the remaining sinuses were adequately opened in less than half of included patients. Frontal sinus openings were the least adequately opened with approximately one-quarter of included patients having their frontal sinuses adequately opened.

In general, the sinuses that were graded as most often adequately complete by the surgeons represent sinuses that are part of the initial and foundational steps in classic functional endoscopic sinus surgery [13]. On the other hand, endoscopic surgery of the sphenoid and frontal sinuses can be regarded as more complex with a higher surgical risk [14]. This can be particularly true in AERD patients who have severe inflammatory disease which may make the surgical fied more difficult to navigate when performing ESS. While middle turbinate resection is not standard of normal ESS, it is a consideration in revision surgery, especially with AERD patients. While it is impossible to know why most patients had incomplete ESS prior to referral to our centre, it is possible that surgical difficulty may have been a contributing factor. Another possible cause of lack of completeness may be related to more intraoperative bleeding in AERD patients potentially leading to early termination of surgery. However, it is also possible that some of these unopened sinuses did not have significant sinus disease at initial presentation and previous surgeons may not have had the same surgical philosophy or intent of performing “complete” sinus surgery during the initial ESS.

In general, the agreement scores across all the sinuses were similar, ranging from fair agreement to moderate agreement [12]. The agreement when all sinus agreement scores were averaged was moderate. Interestingly, the total score mean agreement was the only agreement metric that received “slight agreement” categorization. This is likely due to the nature of such analyses as for all individual measurements the agreement scores were calculated using binary values (0 or 1) hence the variation between graders would only be either 0 or 1. Whereas for the total scores, the variation could be between 0 and 14 leaving greater opportunity for worse agreement scores. Despite this, the average total scores across all patients for each surgeon grader were 6.6, 7.1, 6.4 and 6.4. Variations between the surgeons may represent individual training, personal preference, and institutional differences.

The case for complete sinus surgery in patients with CRS relies on the fact that the mainstay of medical treatment in this disease is ongoing topical therapy (i.e. saline and nasal steroid irrigations). From a delivery standpoint, prior studies have demonstrated an improved penetration of topical delivery when all sinuses have been completely opened, with little to no penetration of the frontal and sphenoid sinuses in the unoperated state [15]. Furthermore, in this disease of epithelial and immune barrier dysfunction, it has been postulated that complete sinus surgery lessens the inflammatory load from the sinuses which may be driving the ongoing cycle of mucosal inflammation [16]. From an outcome perspective, full-house revision ESS can improve patient symptoms, mucosal swelling and LM scores [17]. Hwang et al. recently evaluated the correlation between extent of sinus surgery and postoperative outcomes [18].They demonstrated that patients who had appropriate extent of surgery in concordance with the severity of their disease had the best long term outcomes (greatest reduction in SNOT-22 scores) [18]. DeConde et al. [19] found that complete sinus surgery improved postoperative SNOT-22 scores in CRS patients compared with more targeted, limited surgery, although this difference did not reach the minimal clinically important difference threshold. In other words, patients with more severe disease may benefit from more complete sinus surgery. Given that AERD patients tend to have higher LM scores—on average 20, as demonstrated by Grose et al. [20], it can be hypothesized that these patients could benefit from more comprehensive surgery to match their disease burden. Some authors have advocated for even more extended procedures such as the endoscopic modified Lothrop procedure (EMLP) in AERD patients. Outcome studies in AERD patients who have undergone the EMLP have demonstrated a 22.5% revision surgery rate, which is slightly less than the published rate of 26.2% for complete ESS in this same population [20, 21]. Despite this, additional studies need to be undertaken to determine if additional extended procedures truly result in improved and more durable outcomes.

Biologic therapies such as dupilumab, mepolizumab [21] as well as AD are beneficial for AERD patients [22, 23]. Nonetheless, they do not come without potential costs and risks. For example, for biologic treatment, risks have been described such as: atopic dermatitis, injection site infections, and most commonly conjunctivitis which can occur in 38% of patients [24]. Adverse effects of AD risks can include: major bleeding, gastritis, and rashes with a relative risk of 4.39 [25]. Additionally, reducing the patient’s inflammatory load increases the effectiveness of AD. Hence, given the aforementioned possible additional benefit of complete surgery for AERD patients, assessment of surgical completeness prior to initiating adjuvant treatments may be prudent. With these assessments, surgeons may begin to have conversations with their patients who have had prior surgery regarding surgical completeness. This may allow for collaborative decision making with the patient’s perception of their disease and surgeon regarding the need for revision surgery versus biologic therapy.

This study has certain limitations. Primarily, it is retrospective and was limited by unavailability of certain patient data leading to some patients being excluded from the study. Additionally, the agreement between the four rhinology surgeons was ‘moderate’ as opposed to ‘substantial’ or ‘almost perfect’, hence there is evidence of variability between the surgeons. Nonetheless, the trends between the different sinuses and completeness remained consistent between the surgeons. More so, surgical completeness was only being measured by one factor—postoperative imaging. Furthermore, the scores used were binary and perhaps more granular data could more accurately reflect extent of prior surgery. Finally, we were not able to calculate intra-rater agreement as well as compare the scoring system used with newer scoring systems, such as the ACCESS score [26]. The LM score is very similar to the ACCESS score as it addresses each sinus cavity (maxillary, anterior ethmoid, posterior ethmoid, sphenoid and frontal sinus). The LM score has the additional granularity of scoring adequacy of uncinectomy and whether a middle turbinate resection was completed. As noted in our methodology, the rhinologists would only score a LM 1 if minimal or no additional bony dissection would be required for the opening of a particular sinus cavity. This is in essence equivalent to a score of 0 on the ACCESS score (i.e. no additional dissection is required). While we do not have the additional granularity of scoring "partial surgery" (i.e. ACCESS score of 1) for a particular sinus cavity, we believe that the LM score provides a similar overall assessment of the degree and extent of surgery that has been previously performed. In fact, one could argue that there is no utility in only partial opening of a particular sinus (i.e. partial opening of the posterior ethmoid) if the goal of “adequate” or full-house ESS in severe inflammatory CRS (i.e. AERD in our study) is to provide maximal ventilation and access for topical steroid delivery.

Future research should continue to study objective and prospective ways to define what constitutes adequate surgical therapy for patients with AERD. Clearly, incorporating newer scoring systems such as the ACCESS score will be important to enhance cross-study comparisons. Most importantly, studying long-term outcomes of patients who may have had different degrees of sinus surgery will help define what constitutes the optimal surgical environment for this difficult endotype of CRS with nasal polyps. The heterogeneity of sinus surgery is clearly an area which needs further investigation and may be an important variable in the management of patients with AERD.

## Conclusion

In this study, we found that surgical openings from prior surgery in AERD patients were mostly deemed incomplete by four independent sinus specialists. Maxillary antrostomy and uncinectomy were the steps most adequately completed. It remains to be seen whether additional “complete” sinus surgery would provide greater disease control in AERD patients.

## Data Availability

The data used during the current study are available from the corresponding author on reasonable request.
